# Parental Phubbing and Adolescent Depressive Symptoms during COVID-19: A Serial Meditating Model

**DOI:** 10.3390/bs13050371

**Published:** 2023-04-30

**Authors:** Wencheng Yang, Lu Tang, Xiangping Shen, Gengfeng Niu, Xiaohan Shi, Siyu Jin, Yumang Zhang, Zihui Yuan

**Affiliations:** 1 Key Laboratory of Human Development and Mental Health of Hubei Province, School of Psychology, Central China Normal University, Wuhan 430079, China; 2 Key Laboratory of Adolescent Cyberpsychology and Behavior (CCNU), Ministry of Education, Wuhan 430079, China; 3 Lingui Middle School, Guilin 541100, China; 4 Collaborative Innovation Center of Assessment toward Basic Education Quality, Central China Normal University Branch, Wuhan 430079, China; 5 Center for Research on Internet Literacy and Behavior, Central China Normal University, Wuhan 430079, China

**Keywords:** parental phubbing, parent–child relationship, self-concept clarity, depressive symptoms

## Abstract

Background: During the COVID-19 pandemic, depressive symptoms, a common emotional problem among adolescents, have become more prominent. Regarding the influencing factors of adolescent depressive symptoms, it is widely accepted that parents’ problematic cellphone use around the family (specifically parental phubbing) is a strong predictive factor for the development of depressive symptoms among adolescents. Notably, the COVID-19 pandemic caused a sharp increase in the number of individuals with depressive symptoms, and the negative consequences of parental phubbing and depressive symptoms might have been exacerbated. Accordingly, this study aimed to examine the association between parental phubbing and adolescent depressive symptoms as well as their underlying mechanism. Method: To test our hypotheses, we conducted an offline/online survey with 614 adolescents in Central China from May to June 2022, which corresponded to a period of strict lockdowns in some areas due to the outbreak of the Omicron variant. The participants completed a set of measures, including a technology interference questionnaire, a parent–child relationship scale, a self-concept clarity scale, and the depressive symptoms scale. Results: Parental phubbing was positively associated with adolescent depressive symptoms; the parent–child relationship and self-concept clarity could independently mediate this relationship; and the parent–child relationship and self-concept clarity were also serial mediators in this association. These findings extend previous research by highlighting the impact of parental technology use on their children and the underlying mechanism explaining adolescent depressive symptoms. They provide practical recommendations for parents to prioritize fostering a positive family environment and minimizing phubbing behaviors to enhance adolescent development, particularly in the context of the COVID-19 pandemic.

## 1. Introduction

During the COVID-19 pandemic, depression has become an increasingly prevalent mental health concern for society [1], especially adolescents. Accordingly, depression screening for secondary school students has been an important part of public health programs in China since 2021. A large Chinese sample study revealed that the prevalence of depression was as high as 13.7%, and the reports of depressive symptoms were more common during the COVID-19 pandemic. Depressive symptoms may serve as a risk factor for a range of adverse outcomes, including depressive disorders, self-injury, suicide, and mental and physiological disorders [2]. In particular, adolescents are at a higher risk of developing mood problems [3]; a study found that adolescents with severe depressive symptoms has reached 20% [4]. Remarkably, the COVID-19 pandemic has caused a sharp increase in individuals with depressive symptoms. Based on this, the factors influencing adolescent depressive symptoms have received extensive attention, with social–environmental factors such as social support and parent–child relationships being particularly prominent [5,6]. Meanwhile, with the development of information technology, smartphones have become an integral part of individuals’ lives. According to Global Mobile Statistics, the current global smartphone user population exceeds six billion individuals. China is one of the countries with the highest number of smartphone users [7]. Despite the convenience and comfort of mobile phones, they also pose the risk of falling into the “trap” of problematic use. Particularly during the current stage of the pandemic, new infections are still emerging in many regions. Many Chinese cities have implemented strict lockdowns in response to the outbreak of the Omicron variant [8]. Against this background, the impact of cell phone use on interpersonal relationships and adolescents’ self-development, especially within the family system [9], has become increasingly prominent. Studies have found that parents’ problematic cellphone use (specifically parental phubbing) can increase depressive symptoms in children; however, the internal mechanism of this relationship (i.e., how parental phubbing is linked to adolescent depressive symptoms) in the context of the COVID-19 pandemic has not been well studied. Based on the above evidence, to shed light on potential methods for preventing the development of severe depressive symptoms and clinical depression in adolescents, this study further aimed to examine the association between parental phubbing and adolescent depressive symptoms, especially with respect to its underlying mechanisms.

### 1.1. Parental Phubbing, Parent–Child Relationship, and Depressive Symptoms

In recent years, phubbing has garnered significant attention, driven by the proliferation of Internet applications and the portability of mobile devices. It refers to the phenomenon of excessively engaging in cell phone use, resulting in a lack of attention towards others or excluding them from social interactions. [7]. As an unhealthy phone use behavior and social phenomenon, phubbing has many negative effects, including detrimental impacts on basic psychological satisfaction. It also directly impairs the quality of intimate relationships (e.g., the parent–child relationship) [10,11,12]. Phubbing occurs in a variety of social settings, with the family setting being one of the most important [11,12]. Parental phubbing occurs when parents focus excessively on their phones and neglect their children in their daily interactions [13]. Parental phubbing is significantly related to adolescent depressive symptoms [12,13]. However, studies on the underlying mechanisms of the relationship between parental phubbing and depressive symptoms in adolescents are limited, especially in the context of the pandemic.

During the COVID-19 pandemic, there has been a mounting concern regarding the family atmosphere, particularly in relation to family relationships. Family systems theory, which utilizes ‘system’ to comprehend the role of families in children’s social and emotional development, stresses the significance of dynamic transactions across multiple levels of family systems and highlights the parent–child relationship’s important influence on adolescent development. Based on this theory, a positive parent–child relationship promotes improved psychological health [14,15,16]. Concerning parental phubbing, the ecological techno-subsystem theory proposes the ecological techno-subsystem as a microsystem dimension that includes children’s interactions with various technologies in their immediate environment [17,18,19]. That is, mobile phone use can affect adolescent adaptation differently by affecting their immediate living environment (e.g., the parent–child relationship) [18,19]. Parental phubbing, as a form of a problematic cell phone use behavior, may cause successive psychological and behavioral problems. This neglect may induce a perception of social exclusion and reduce face-to-face interactions among affected children in real life, leading to interpersonal difficulties [11,20]. In addition, recent studies revealed a negative association between phubbing and a positive parent–child relationship [21,22].

Additionally, a positive parent–child relationship is critical in maintaining adolescents’ mental health [23], especially during the COVID-19 pandemic. Based on attachment theory, a good parent–child relationship can provide children with warmth and social support [24]. Additionally, it was found that positive parent–child relationships could reduce adolescent depressive symptoms [25], but conflicts between parents and children (a poor parent–child relationship) are positively associated with children’s depressive symptoms [26]. Thus, parental phubbing may precipitate adolescent depressive symptoms by deteriorating the parent–child relationship. Based on this reasoning, we hypothesized that parental phubbing could affect depressive symptoms through the mediating role of the parent–child relationship (H1).

### 1.2. The Potential Mediating Roles of Self-Concept Clarity

Self-development is one of the most critical mental health issues in adolescence [27], and self-concept clarity is an essential indicator of self-growth. Self-Concept Clarity (SCC) refers to the extent to which an individual’s self-concept is clearly and confidently defined, as well as its temporal stability and internal consistency [28]. Based on the cognitive theory of depression [29], self-concept clarity has good adaptive value for individuals, and low self-concept clarity is a risk factor for depression [30]. Self-concept clarity develops gradually and is influenced by many environmental factors, including peer relationships and Internet use, and especially by family factors, such as parental phubbing.

According to the sociometer theory [31], self-evaluation is highly sensitive to cues of social rejection. Increased parental phubbing may cause adolescents to develop negative self-evaluations, thus impairing their self-concept clarity. Moreover, low self-concept clarity is also an essential mediating mechanism in the relationship between environmental factors and depressive symptoms [32], so we hypothesized that self-concept clarity might have a potential mediating role in the relationship between parental phubbing and adolescent depressive symptoms (H2). Moreover, interpersonal relationships are also an important influencing factor, especially the parent–child relationship. For example, one study found that the parent–child relationship serves as a crucial internal mechanism in the relationship between parental phubbing and self-concept. [21]. In addition, empirical studies have indicated that a poor parent–child relationship could adversely affect self-concept clarity [33]. Based on this reasoning, the present study further hypothesized that parental phubbing was associated with depressive symptoms through the serial mediation effect of the parent–child relationship and self-concept clarity (H3).

**Figure 1:**
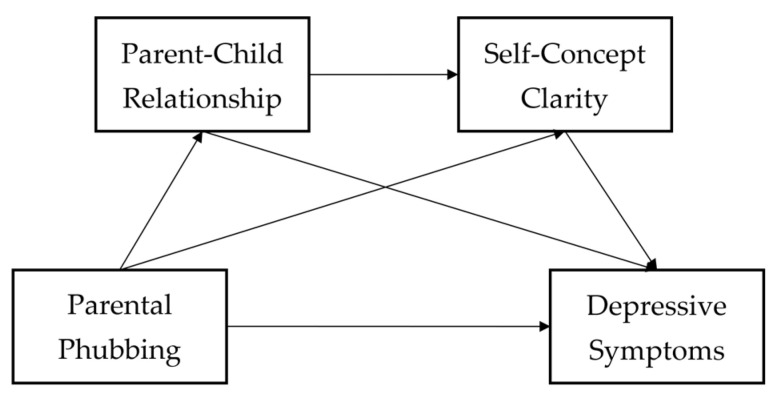
The hypothesized serial mediation model.

In summary, against the background of the COVID-19 pandemic and the reality of adolescents’ lives and within the perspective of family systems theory and the ecological techno-subsystem theory, this study constructed a serial mediation model in order to explore the association between parental phubbing and adolescent depressive symptoms as well as its mechanism, namely, the mediating role of the parent–child relationship and self-concept clarity. The following hypotheses were proposed (the hypothesized model is presented in Figure 1):

**H1.**  *Parental phubbing is positively correlated with adolescent depressive symptoms through the mediating role of the parent–child relationship*.

**H2.**  *Parental phubbing is positively correlated with adolescent depressive symptoms through the mediating role of self-concept clarity*.

**H3.**  *The parent–child relationship and self-concept clarity act as serial mediators between parental phubbing and adolescent depressive symptoms*.

## 2. Materials and Methods

### 2.1. Participants

We used convenience sampling and recruited 614 students from two middle schools in central China. Ultimately, 22 participants dropped out of the study for personal reasons, while 592 students (310 females; the average age was 14.92 *±* 1.17 years old) completed the survey questionnaire. 

### 2.2. Procedure

We conducted this cross-sectional survey from May to June 2022. First, standardized instructions were provided, followed by the distribution of paper-pencil questionnaires to each participant. The surveys were conducted in class. At the outset of the study, most participants attended classes remotely (from home), so they completed an online survey. To compensate for their participation, each participant received a small gift or an electronic red envelope (approximately 0.5 US dollars). This study was approved by the Ethical Committee for Scientific Research at the authors’ university, and all participants provided written consent.

### 2.3. Measurement

Parental Phubbing. The 6-item questionnaire developed by McDaniel and Coyne [34] was adopted to measure the frequency of adolescents’ parental phubbing (e.g., “During a typical mealtime that my parents and I spend together, my parents pull out and check their cell phones.”). Participants were asked to evaluate the frequency of each item on a 5-point scale ranging from 1 (never) to 5 (all the time); the items’ scores were averaged to generate the participants’ scores, where higher scores indicated higher levels of parental phubbing. In the present study, the results of the validation factor analysis showed good fits: χ^2^/*df* = 4.17, RMSEA = 0.06, CFI = 0.94, and TLI = 0.92; in our study, Cronbach’s alpha was set to 0.87.

Parent–Child Relationship. To assess the relationship between adolescents and their parents, we adopted the Chinese version of the parent–child relationship scale [35]. The scale contains 16 items measuring participants’ relationships with their mother/father (e.g., you and your mother/father understand each other well); participants were required to respond using a 3-point scale ranging from 1 (never) to 3 (often), where higher averaged scores indicated a better relationship with parents. The Cronbach’s alpha was 0.92.

Self-Concept Clarity. We adopted the Chinese version of the self-concept clarity scale to measure participants’ self-concept clarity (e.g., “I have a clear sense of who I am and what I am”) [28,36]. The scale included 12 items, and participants were required to respond using a 5-point scale ranging from 1 (strongly disagree) to 5 (strongly agree). Then, we calculated the average score to generate the participants’ scores, where higher scores indicated better self-concept clarity. The Cronbach’s alpha was 0.88.

Depressive symptoms. We adopted the Chinese version of the 20-item Center for Epidemiological Studies Depression Scale (CES–D) [37] to measure participants’ depressive symptoms (e.g., “I feel that everything takes a lot of effort.”). Participants were asked to assess how often they had been bothered by each item over the last week on a 4-point scale; the scores of all the items were averaged to generate the participants’ scores, where higher scores indicated more severe depressive symptoms. The Cronbach’s alpha was 0.93.

### 2.4. Statistical Analysis

We adopted SPSS 28.0 and SPSS macro PROCESS (http://www.afhayes.com, accessed on 10 July 2022) to analyze the data. First, we determined descriptive statistics and correlations. Subsequently, we used Hayes’ [38] SPSS macro PROCESS (Model 6) to test the hypothesized model, which can test both the mediating effect and the serial mediation effect in one model. Moreover, we used 5000 bias-corrected bootstrapped resampling to estimate the mediating effect’s 95% confidence interval (the effect is considered significant if the 95% confidence interval values do not include zero). Considering gender and age were associated with depressive symptoms [39], they were included as covariates.

## 3. Results

### 3.1. Descriptive and Correlations Statistics

As shown in Table 1, all the main research variables were significantly correlated with each other. Parental phubbing was negatively correlated with the parent–child relationship and self-concept clarity, while it was positively correlated with depressive symptoms. Moreover, both the parent–child relationship and self-concept clarity were negatively correlated with depressive symptoms, while the parent–child relationship was positively correlated with self-concept clarity.

### 3.2. The Serial Mediation Model Analysis

**Figure 2:**
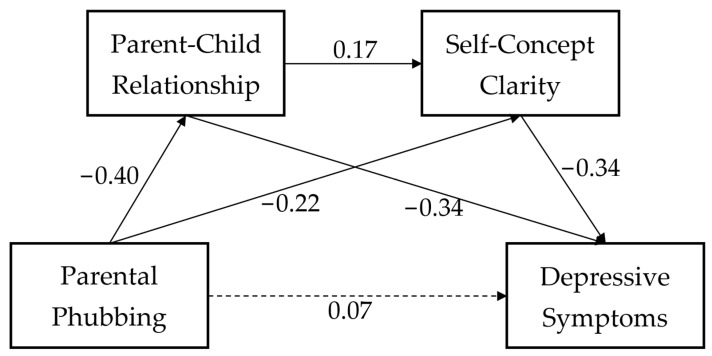
The serial mediation model.

First, we used simple linear regression to examine the association between parental phubbing and depressive symptoms. After controlling for gender and age, the results showed that parental phubbing was positively associated with depressive symptoms (β = 0.12, *p* < 0.001). Then, we used SPSS macro PROCESS to examine our hypothesized serial mediation model. As shown in Table 2, after controlling for gender and age, it was found that parental phubbing was negatively associated with the parent–child relationship (β = −0.40, *p* < 0.001) and self-concept clarity (β = −0.22, *p* < 0.001); the parent–child relationship was positively associated with self-concept clarity (β = 0.17, *p* < 0.01) and negatively related to depressive symptoms (β = −0.34, *p* < 0.001); and self-concept clarity was negatively related to depressive symptoms (β = −0.34, *p* < 0.001). When both the parent–child relationship and self-concept clarity were included in the regression model to predict depressive symptoms, the association between parental phubbing and depressive symptoms was not significant. The serial mediation model is shown in Figure 2.

Then, to further test and calculate the mediating effects found in our serial mediation model, we used a bootstrap program. We found that all three mediating paths did not include 0 in their 95% confidence intervals, indicating that each mediating path was significant. Thus, all hypotheses were supported. The effects and relative values for each mediating path are presented in Table 3.

## 4. Discussion

Based on the background of the COVID-19 pandemic and the social fact that digital applications (represented by mobile phones) are increasingly being integrated into people’s daily lives, we constructed a serial mediation model to examine the association between parental phubbing and adolescent depressive symptoms and its potential underlying mechanism. The results showed that parental phubbing was positively correlated with adolescent depressive symptoms; further regression analysis also showed that parental phubbing had a significant positive predictive effect on depressive symptoms. Moreover, the results showed the mediating roles of the parent–child relationship and self-concept clarity in this association. Specifically, this indirect effect consists of three paths: parental phubbing → the parent–child relationship → depressive symptoms; parental phubbing → self-concept clarity → depressive symptoms; and parental phubbing → the parent–child relationship → self-concept clarity → depressive symptoms. Interestingly, when the parent–child relationship and self-concept clarity were entered into the regression model simultaneously, parental phubbing did not directly predict adolescent depressive symptoms. This study may expand previous theories and studies and have significant theoretical and practical implications.

### 4.1. The Mediating Role of Parent–Child Relationship

In accordance with previous studies [10,12], our results found that parental phubbing is positively associated with adolescent depressive symptoms. This finding further emphasizes the influence of parents’ problematic phone use around their family and has important implications for adolescents’ healthy development during the COVID-19 pandemic. Specifically, parents are one of the most influential actors in guiding adolescents’ mental health and social adjustment [40,41]. With the increasing integration of digital technology into daily life, subsequent technology interference affects the typical family atmosphere and function and brings a novel form of parental neglect (parental phubbing) [18,19]. Previous studies have found that parental phubbing, as a new form of parental social rejection and neglect, can directly reduce well-being and increase adolescent depressive symptoms [12]. However, we found that when the parent–child relationship and self-concept clarity were entered into the regression model simultaneously, parental phubbing did not directly predict adolescent depressive symptoms. This result further suggested other influential variables in the relationship between parental phubbing and psychosocial adjustment [12,42]. Specifically, parental phubbing does not necessarily lead to depressive symptoms, and the after-effect of parental phubbing on the parent–child relationship is a significant factor.

As hypothesized, parental phubbing was positively associated with adolescent depressive symptoms through the mediating effect of the parent–child relationship. This result is consistent with previous studies [23,26] and further indicates that the parent–child relationship is a crucial factor in the negative adaptive consequences of parental phubbing. A good parent–child relationship plays a significant role in the development and adaptation of an adolescent [9,16]. To better cope with the impact of the COVID-19 pandemic, it is even more crucial to create a good family environment and parent–child relationship for adolescents. However, parents’ excessive attention to cell phones during parent–child interactions can reduce the quality of parent–child interactions and make children feel negative sensations of neglect, both of which can increase the risk of depressive symptoms. This result fits well with and further extends the basic view of the ecological techno-subsystem theory and family systems theory [15,16,17,18], which contend that information technology (e.g., the internet and mobile phones) has become an integral part of family life which might interact with family dynamics (e.g., family members’ relationships) and subsequently affect individuals’ development. Furthermore, parental phubbing is a negative social interaction that can damage a relationship’s quality and social interaction, resulting in a deteriorated parent–child relationship [22]. Meanwhile, studies have found that improper behavior of parents often results in parent–child conflicts and the deterioration of the parent–child relationship’s quality, thereby weakening family functioning and cohesion [12,35]. Previous studies found that the COVID-19 pandemic has significantly changed people’s lifestyles, including by precipitating decreased physical activity and increased reliance on online activities such as social media [43,44]. These lifestyle changes may contribute to an increase in phubbing behavior, leading to more family issues. Furthermore, the pandemic has caused Chinese adolescents to spend more time studying and playing video games online at home. As parents spend more time with their children, phubbing behavior and its adverse effects may become more pronounced. Thus, parental phubbing could lead to adolescent depressive symptoms by deteriorating the parent–child relationship.

### 4.2. The Mediating Role of Self-Concept Clarity

At the same time, the results further revealed the independent mediating effect of self-concept clarity and the serial mediating effect of the parent–child relationship → self-concept clarity in the relationship between parental phubbing and adolescent depressive symptoms. In accordance with previous findings, our results further conformed that poor self-concept clarity was a significant predictor of depressive symptoms, especially for adolescents [34]. Adolescence is a crucial developmental stage for families and society and is a critical period for self-development [37]. Adolescents with higher self-concept clarity are more likely to adopt positive cognitive reappraisal strategies and take positive actions to face adverse events, whereas individuals with lower levels of self-concept clarity have a higher susceptibility to negative events [29,44]. In addition, low self-concept clarity is associated with greater confusion regarding identity processes, ambivalence [45], negative self-perceptions, and negative emotional experiences [31,46]; especially during the COVID-19 pandemic, these negative effects may be more pronounced. Thus, individuals with low self-concept clarity are at greater risk for developing depressive symptoms. At the same time, adolescents’ self-concept clarity develops gradually [30]. In the context of the COVID-19 pandemic, adolescents are sensitive to the influence of their social environment and families and are more likely to be influenced by negative parent–child interactions (parental phubbing) [47]. Thus, parental phubbing could lead to adolescent depressive symptoms by reducing adolescents’ self-concept clarity.

In addition, the parent–child relationship is also closely associated with self-concept clarity [30]. In good parent–child relationships, active parental listening and support increase adolescents’ internal reflection and self-talk, which, in turn, promotes the development of self-concept clarity [30]. On the other hand, Chinese adolescents grow up in a collectivist culture, and most of them often tend to develop an interdependent self in relation to their parents, in contrast to those raised in individualist cultures. [48], so their self-concepts are more likely to be influenced by parent–child interactions. As a negative phenomenon in interpersonal interactions, parental phubbing can lead to a negative self-concept in adolescents, which increases the risk of developing depressive symptoms. Furthermore, according to the self-processing model of depressive symptoms [29], when there are problems in the parent-child relationship, adolescents may experience insufficient access to the developmental resources that the relationship could provide, which in turn increases the risk of depressive symptoms. Thus, impaired parent–child relationships resulting from parental phubbing can impair self-concept clarity and further induce depressive symptoms.

### 4.3. Limitations of the Study

Some of this study’s limitations should be acknowledged. First, though based on theoretical and empirical evidence, cross-sectional designs cannot be used to make causal inferences. Thus, future studies should focus more on longitudinal, prospective, or experimental methods. In addition, future studies should focus on the influencing factors of parental phubbing and provide theoretical support for interventions. Second, since phubbing is sometimes unavoidable due to the widespread use of cell phones in daily life, future studies should pay more attention to individual differences, especially the positive factors that can play a protective role in phubbing situations. Third, the effects of parental phubbing on depressive symptoms may differ in different cultures. Future studies should consider including diverse participants to explore potential cultural differences.

## 5. Conclusions

In summary, the present study showed that parental phubbing is associated with adolescent depressive symptoms during the COVID-19 pandemic. We also found significant mediating effects of both the parent-child relationship and self-concept clarity, as well as a serial mediating effect. This study emphasizes the potential damage problematic cell phone use can inflict on family relationships and functioning. In addition, it emphasizes the importance of parents reducing phubbing behavior. Moreover, these findings stress that parents should focus on creating a positive family atmosphere to help adolescents build stable and clear self-concepts.

Although it has limitations, this study has some theoretical and practical implications. First, the parent–child relationship and self-concept clarity were introduced to examine the mechanism underlying the relationship between parental phubbing and adolescent depressive symptoms, which will broaden family systems theory and shed light on how adolescents’ self-development is associated with poor parental behavior within a family. Second, our results enrich previous studies, contributing to a better understanding of how parental phubbing is related to adolescent depressive symptoms.

In terms of practical implications, this study provides some empirical evidence and guidance for parent–child family education, especially with respect to shaping a good family atmosphere under the current COVID-19 pandemic. First, parents should acknowledge the dark side of modern technology (especially phubbing) and its effects on family functions and adolescents’ adaptation. Parents should try to have open and honest discussions with their children about technology use and how it may affect family dynamics [49]. They should also establish clear rules and boundaries with respect to technology use during family time, such as implementing designated “tech-free” times or areas in the house. Furthermore, this study suggests that parents should prioritize spending quality time with their children, particularly through activities that promote face-to-face interaction and socialization. This can include activities such as family game nights, cooking together, or engaging in physical exercise. Additionally, parents should act as models of healthy technology use behavior [24], e.g., by limiting their own screen time and practicing mindfulness when using technology. Overall, this study underscores the importance of maintaining a healthy balance between technology use and family time, particularly during the COVID-19 pandemic, wherein online activities have become more prevalent.

## Figures and Tables

**Table 1:** Means, standard deviations, and correlation results.

Variables	*M* (*SD*)	1	2	3	4
1. Parental Phubbing	2.09 (0.79)	1			
2. Parent–child relationship	2.31 (0.38)	−0.41 **	1		
3. Self-concept clarity	3.06 (0.99)	−0.29 **	0.27 **	1	
4. Depressive symptoms	1.76 (0.46)	0.46 **	−0.50 ***	−0.47 **	1

** *p* < 0.01; *** *p* < 0.001.

**Table 2:** Testing the serial mediation effect.

Outcome	Predictors	*R* ^2^	*F*	*β*	*t*	LLCI	ULCI
PCR		0.22	27.12 ***				
	Gender			−0.14	−1.32	−0.35	0.07
	Age			−0.18	−3.69 ***	−0.26	−0.09
	PP			−0.40	−7.66 ***	−0.50	−0.30
SCC		0.13	10.65 ***				
	Gender			−0.25	−2.21 *	−0.47	−0.03
	Age			0.01	0.26	−0.08	0.11
	PP			−0.22	−3.59 ***	−0.33	−0.10
	PCR			0.17	2.81 **	0.05	0.30
DS		0.38	36.25 ***				
	Gender			0.16	1.61	−0.03	0.34
	Age			0.07	1.69	−0.11	0.14
	PP			0.07	1.34	−0.03	0.17
	PCR			−0.34	−6.56 ***	−0.45	0.24
	SCC			−0.34	−6.91 ***	−0.43	0.24

* Gender: = male and 1 = female; PCR = parent–child relationship, PP = parental phubbing, DS = depressive symptoms; bootstrap sample size = 5000; LL = lower limit, CI = confidence interval, UL = upper limit; * *p* < 0.05, ** *p* < 0.01, and *** *p* < 0.001; all the variables in the analysis were standardized.

**Table 3:** Total, indirect effects of parental phubbing (X) on depressive symptoms (Y) through parental–child relationship (M1) and self-concept clarity (M2).

	Indirect Effect	LLCI	ULCI	Relative Value
X → M1 → Y	0.20	0.09	0.20	65%
X → M2 → Y	0.15	0.02	0.13	48%
X → M1 → M2 → Y	0.06	0.01	0.05	19%

## Data Availability

The data of this study are available from the corresponding author upon reasonable request.
